# Latent Cytomegalovirus Infection and Previous Capsular Polysaccharide Vaccination Predict Poor Vaccine Responses in Older Adults, Independent of Chronic Kidney Disease

**DOI:** 10.1093/cid/ciab078

**Published:** 2021-03-16

**Authors:** Nadezhda Wall, Alexandra Godlee, Daniel Geh, Charlotte Jones, Sian Faustini, Ruth Harvey, Rebecca Penn, Dimitrios Chanouzas, Peter Nightingale, Matthew O’Shea, Alex Richter, Paul Moss, Adam Cunningham, Lorraine Harper

**Affiliations:** 1 Institute of Clinical Sciences, College of Medical and Dental Sciences, University of Birmingham, Edgbaston, Birmingham, UK; 2 Institute of Immunology and Immunotherapy, College of Medical and Dental Sciences, University of Birmingham, Edgbaston, Birmingham, UK; 3 National Institute for Biological Standards and Control, Potters Bar, UK; 4 Institute Translational Medicine, University Hospitals Birmingham NHS Foundation Trust, Edgbaston, Birmingham, UK

**Keywords:** chronic kidney disease, vaccination, immunodeficiency, cytomegalovirus, adaptive immunity

## Abstract

**Background:**

Patients with chronic kidney disease (CKD) are more prone to severe infection. Vaccination is a key strategy to reduce this risk. Some studies suggest vaccine efficacy may be reduced in patients with CKD, despite preserved maintenance of long-term responses to some pathogens and vaccines. Here, we investigated immune responses to 2 vaccines in patients with CKD to identify predictors of immunological responsiveness.

**Methods:**

Individuals >65 years old, with or without nondialysis CKD (n = 36 and 29, respectively), were vaccinated with a nonadjuvanted seasonal influenza vaccine (T-dependent) and Pneumovax23 (23-valent pneumococcal polysaccharide [PPV23], T-independent). Humoral responses were measured at baseline, day 28, and 6 months. Lymphocyte subset and plasma cell/blast analyses were performed using flow cytometry. Cytomegalovirus (CMV) serotyping was assessed by enzyme-linked immunosorbent assay.

**Results:**

Only modest responsiveness was observed to both vaccines, independent of CKD status (25% adequate response in controls vs. 12%–18% in the CKD group). Unexpectedly, previous immunization with PPV23 (median 10-year interval) and CMV seropositivity were associated with poor PPV23 responsiveness in both study groups (*P* < .001 and .003, respectively; multivariable linear regression model). Patients with CKD displayed expanded circulating populations of T helper 2 and regulatory T cells, which were unrelated to vaccine responses. Despite fewer circulating B cells, patients with CKD were able to mount a similar day 7 plasma cell/blast response to controls.

**Conclusion:**

Patients with nondialysis CKD can respond similarly to vaccines as age- and sex-matched healthy individuals. CKD patients display an immune signature that is independent of vaccine responsiveness. Prior PPV23 immunization and CMV infection may influence responsiveness to vaccination.

**Clinical Trials Registration**. NCT02535052

## Teaser

Patients with nondialysis Chronic Kidney Disease (CKD) can mount similar responses to vaccination as controls. Subtle changes in lymphocyte phenotype are seen in CKD when controlled for effect of Cytomegalovirus (CMV). Latent CMV infection and previous PPV23 vaccination are associated with poorer vaccine responses.

**(See the Editorial Commentary by Peppa and Reeves on pages e890–1.)**

Chronic kidney disease (CKD) is common, affecting approximately 8% of the UK population, and prevalence increases with advancing age [1]. Patients with CKD have an elevated risk of infection, which increases with disease severity and accounts for 20% of mortality [2, 3]. The etiology of this increased risk is not fully understood. Vaccination is an obvious potential preventive strategy, but lower rates of seroconversion and impaired maintenance of humoral vaccine responses are reported in CKD [4, 5]. However, humoral responses to historically encountered antigens, such as tetanus or diphtheria toxoids, are similar between patients with CKD and age-matched controls [6]. Moreover, antibody function (eg, killing of *Salmonella enterica*) can be maintained in patients with CKD [6].

Other factors are associated with increased risk of infection, including age [7]. Previous exposure to plain polysaccharides may cause hyporesponsiveness to subsequent vaccination; hence, current guidance advises revaccination after 5 years [8]. Furthermore, previous infection with cytomegalovirus (CMV) can also negatively affect vaccine responses in healthy older adults [9–12], although this has not been examined in CKD. Therefore, multiple other factors could influence vaccine responsiveness in patients with CKD.

In this study, we evaluated responsiveness to vaccination in a carefully matched cohort of older patients with and without nondialysis CKD. We found only subtle differences between the study groups. CMV and previous 23-valent pneumococcal polysaccharide (PPV23) vaccination emerged as the strongest predictors of vaccine responsiveness, rather than CKD. As such, appreciation of both disease-specific and environmental immune-modulating factors is needed to optimize vaccination strategy in older susceptible patient populations.

## METHODS

### Study Design

Patients with CKD and healthy controls were recruited to a prospective observational cohort study (clinicaltrials.gov ID:NCT02535052) between June 2015 and January 2018 at University Hospitals Birmingham NHS Foundation Trust (UHBFT), Birmingham, UK. Edgbaston Research Ethics Committee approved the study (ref:15/WM/0057). All participants gave written informed consent.

Subjects were aged ≥65 years, without immune-mediated disease or malignancy within the preceding 5 years. Patients not requiring dialysis, with laboratory estimated glomerular filtration rate (eGFR) 15 to 60 mL/min were recruited from UHBFT CKD outpatient clinics. Controls were relatives of patients or from the wider community, with eGFR >60 mL/min. Demographics and baseline clinical information were collected from electronic clinical records and vaccination history from primary care records. Individuals that had received PPV23 within 5 years were excluded.

Participants were vaccinated with trivalent inactivated influenza (TIV) and PPV23 intramuscularly per manufacturer instructions (PPV23; Merck, USA; TIV; Sanofi, France). PPV23 contains capsular polysaccharides for 23 serotypes of *Streptococcus pneumoniae*. TIV contained hemagglutinin antigens from 2 influenza A strains and 1 B strain, which varied between years based on World Health Organization northern hemisphere recommendations [13].

Peripheral blood was collected at baseline, days 7 and 28, and month 6 after vaccination. Serum was separated from blood and frozen at −20°C. Peripheral blood mononuclear cells (PBMCs) were separated by density gradient centrifugation (Ficoll-Paque PLUS, GE Healthcare) and either used immediately or frozen for batched analysis. Baseline and/or month 6 PBMCs were used for lymphocyte phenotyping analysis and day 7 samples were evaluated for plasma cells/blasts (PC/B).

### Influenza Hemagglutination Inhibition Assay

NIBSC laboratories (Potters Bar, UK) performed the influenza hemagglutination inhibition (HAI) assay using standard methods as previously described [14]. All test sera were tested twice against the relevant vaccine strains from each vaccine season.

### Measurement of Immunoglobulin G to Pneumococcal Polysaccharides

Concentrations of immunoglobulin G (IgG) against 12 PPV23 antigens (1, 3, 4, 5, 6B, 7F, 9V, 14, 18C, 19A, 19F, 23F) were determined in the UHBFT Clinical Immunology Laboratory using a validated multiplex assay, as previously described [15]. To minimize inter-assay variability, samples were processed in batches with all timepoints from an individual included in the same assay.

### Vaccine Response Adequacy Criteria

Adequate responses to influenza hemagglutinin antigens and pneumococcal polysaccharides (PnPS) were defined as the conversion from a nonprotective to protective titer (TIV: HAI ≥40; PPV23: serotype-specific anti-PnPS IgG titer ≥0.35 μg/mL) [13, 16]). If prevaccination titers were protective, responses were considered adequate if a 2-fold (for PPV23) or 4-fold (for TIV) increase in titer was seen at day 28 postvaccination [13, 16]. An adequate response to the vaccine as a whole was defined as an adequate response to 2/3 TIV strains or 8/12 PPV23 serotypes.

### Measurement of CMV-specific IgG

Serum CMV-specific IgG titer was determined using a semiquantitative in-house enzyme-linked immunosorbent assay as previously described [17].

### Immunophenotyping

Flow cytometric phenotyping of lymphocytes was performed using an LSR Fortessa instrument and results analyzed using FACSDiVa software (v8.0; BD Biosciences, UK). Cells were incubated in BD Brilliant staining buffer for 30 minutes with pretitrated volumes of fluorochrome-conjugated antibodies (Supplementary Table 1). Intracellular staining for cytokines and transcription factors was performed using a FoxP3/Transcription Factor Staining Buffer Kit, as per manufacturer instructions (eBioscience, USA). Unstimulated PBMCs were used to set negative gates and fluorescence minus 1 controls were used where required. Representative gating strategies are shown in Supplementary Figures 2, 5, and 7.

All phenotyping was performed using cryopreserved cells, except for surface chemokine receptor staining to define T helper 1 (Th_1_)/T helper 2 (Th_2_)/T helper 17 (Th_17_)/follicular T helper T (Th_fh_) cell CD4 populations, which was done on fresh PBMCs. Th_1_ and Th_2_ CD4 phenotypes were also defined by cytokine production: cryopreserved PBMCs were cultured for 18 hours at 37°C, 5% CO_2_, with 5 µg/mL plate-bound aCD3, soluble aCD28, and aCD49d (Supplementary Table 1) in complete Roswell Park Memorial Institute medium with brefeldin A and monensin (eBioscience, USA).

### Statistics

Statistical analyses were performed using Prism (version 7, GraphPad, USA) and SPSS software (version 24, IBM, USA). Continuous data were tested for normality (using Shapiro-Wilk or Kolmogorov-Smirnoff tests) and groups were compared using parametric (unpaired/paired *t* test, analysis of variance) and nonparametric methods (Mann-Whitney test) as appropriate. Categorical data were compared using Fisher’s exact or χ ^2^ tests. Correlations between continuous data were assessed using Pearson’s and Spearman’s rank tests as appropriate. Multivariate analysis on continuous data (normalized as appropriate) was performed using linear regression modelling. In all statistical analyses, 2-tailed *P* < .05 was considered statistically significant. Bonferroni correction was applied to data with multiple comparisons.

## RESULTS

### Participant Characteristics

Sixty-five individuals were vaccinated: 29 controls and 36 patients with CKD. Four individuals (1 control, 3 patients with CKD) were excluded from the final analysis because they either developed health conditions that met study exclusion criteria (n = 2) or were lost to follow-up (n = 2). Patients with CKD were similar in age and sex as controls, but had greater comorbidity (Table 1). Patients with CKD had a median eGFR of 21 mL/min. Compared with controls, patients with CKD were anemic and had a significantly higher white cell count, neutrophil count, and C-reactive protein (Table 1). The prevalence of latent CMV (defined by CMV IgG seropositivity) and serum levels of CMV-specific IgG in seropositive individuals were not significantly different between disease groups.

**Table 1.** Demographic, Clinical, and Laboratory Parameters of Study Participants

	Controls (n = 28)	CKD (n = 33)	*P* Value
Age, y	74 (11)	75 (11)	.28^a^
Male, n (%)	15 (54)	23 (70)	.29^b^
White ethnicity, n (%)	28 (100)	30 (91)	.24^b^
Current/ex-smoker, n (%)	**10 (36)**	**25 (76)**	**.002** ^**b**^
Received TIV in preceding season, n (%)	24 (86)	30 (91)	.69^b^
Previous PPV23, n (%)	**17 (61)**	**29 (88)**	**.02** ^**b**^
Time from previous PPV23, y	9.9 (5.0)	11.0 (4.2)	.26^a^
Ischemic/hypertensive nephropathy	-	14 (42)	-
Diabetic nephropathy	-	5 (16)	-
Obstructive uropathy	-	2 (6)	-
Other^c^	-	2 (6)	-
Unclear^d^	-	10 (30)	-
Charlson Comorbidity Index	**1 (2)**	**5 (3)**	**<.0001** ^**a**^
DM, n (%)	**1 (4)**	**22 (67)**	**<.0001** ^**b**^
HTN, n (%)	**9 (32)**	**32 (97)**	**<.0001** ^**b**^
CVD (including IHD, CVA/TIA, PVD, CCF, arrhythmias), n (%)	**7 (25)**	**25 (76)**	**.0001** ^**b**^
Number of medications per person	**2 (3)**	**9 (4)**	**<.0001** ^**a**^
Participants with ≥5 medications (polypharmacy), n (%)	**4 (14)**	**32 (97)**	**<.0001** ^**b**^
Hb, g/L	**140 (9)**	**115 (19)**	**<.0001** ^**a**^
WCC, 10^9^/L	**6.0 (2.9)**	**7.2 (3.4)**	**.005** ^**a**^
Neutrophils, 10^9^/L	**3.3 (1.7)**	**4.8 (2.9)**	**<.0001** ^**a**^
Lymphocytes, 10^9^/L	1.6 (1.1)	1.3 (1.0)	.13^a^
Platelets, 10^9^/L	213 (53)	191 (79)	.15^a^
Neutrophil/lymphocyte ratio	**2.1 (0.8)**	**3.8 (2.4)**	**<.0001** ^**a**^
eGFR, mL/min	**77 (16)**	**21 (13)**	**<.0001** ^**a**^
ACR, mg/mmol	**0.5 (1.7)**	**28.8 (153.5)**	**<.0001** ^**a**^
hsCRP, mg/L	**0.8 (1.1)**	**5.1 (5.1)**	**<.0001** ^**a**^
HbA1c, mmol/mol	**36.5 (4.0)**	**50.5 (19.8)**	**<.0001** ^**a**^
CMV seropositive, n (%)	17 (61)	27 (82)	.09^b^
Anti-CMV IgG titer (seropositive only), log_10_ (AU)	2.1 (0.65)	2.2 (0.60)	.74^e^

Median and IQR shown unless stated. Significance level is *P* < .05 (shown in bold).

Abbreviations: ACR, albumin/creatinine ratio; CCF, congestive cardiac failure; CI, confidence interval; CKD, chronic kidney disease; CMV, cytomegalovirus; CVA, cerebrovascular accident; CVD, cardiovascular disease; DM, diabetes mellitus; eGFR, estimated glomerular filtration rate; Hb, hemoglobin; HbA1c, glycated hemoglobin; hsCRP, highly sensitive C-reactive protein; HTN, hypertension; IgG, immunoglobulin G; IHD, ischemic heart disease; PPV23, 23-valent pneumococcal polysaccharide vaccine; PVD, peripheral vascular disease; TIA, transient ischemic attack; TIV, trivalent influenza vaccine; WCC, white cell count.

^a^Mann-Whitney 2-tailed *P*.

^b^Fisher’s exact 2-tailed *P*.

^c^1 lithium toxicity, 1 congenital single kidney.

^d^No clearly defined etiology, but with multiple comorbidities known to cause renal impairment, including HTN and DM.

^e^Unpaired *t* test 2-tailed *P*.

### Inadequate Vaccine Responses Are Observed in Study Participants Irrespective of CKD

At baseline, controls and patients with CKD had similar proportions of individuals with protective HAI titers against any of the 3 TIV influenza strains and protective anti-PnPS IgG concentrations for any of the 12 serotypes tested (Table 2). The proportion of individuals with protective titers to both TIV and PPV23 antigens increased following vaccination in both groups. Whole vaccine responses were modest for both TIV and PPV23, and overall responses were similar in controls and patients with CKD (Table 2). Individuals with adequate vaccine responses to either vaccine at day 28 maintained them at month 6, with no differences between the groups (TIV: 80% [n = 16] controls and 67% [n = 14] patients with CKD; PPV23: 86% [n = 6] controls and 100% [n = 6] patients with CKD). Baseline strain-specific HAI titers and serotype-specific Pn IgG concentrations were similar between the groups (Supplementary Tables 2,3). Patients with CKD demonstrated a similar magnitude of increase in strain-specific HAI titer (defined by the antibody response ratio [ARR]: day 28/day 0 titer) and maintenance of titers (defined by the antibody maintenance ratio: month 6/day 28 titer) as controls following vaccination (Supplementary Table 2). PPV23 vaccination significantly increased serotype-specific anti-PnPS IgG concentrations from baseline to day 28 for all 12 serotypes tested in both controls and patients with CKD and to a similar degree. Maintenance of postvaccination anti-PnPS IgG concentrations (antibody maintenance ratio) was also similar between the groups (Supplementary Table 3). Therefore, CKD status did not influence the magnitude or longevity of these vaccine responses.

**Table 2.** Adequacy of Vaccine Response and Population “Protection” Against Influenza and Pneumococcus in Patients With CKD and Controls Using Clinical Parameters

	Controls (n = 28)			CKD (n = 33)		
	“Protected” at Baseline	“Protected” at Day 28	“Adequate” Response	“Protected” at Baseline	“Protected” at Day 28	“Adequate” Response
Influenza strains A/H1N1	13 (46)	22 (79)	**12 (43)**	14 (42)	22 (67)	**9 (27)**
A/H3N2	17 (61)	23 (82)	**9 (32)**	26 (79)	28 (85)	**6 (18)**
B	6 (21)	10 (36)	**4 (14)**	3 (9)	6 (18)	**3 (9)**
2 of 3 strains	11 (39)	20 (71)	**7 (25)**	12 (36)	21 (64)	**4 (12)**
*Pn serotypes*						
1	12 (43)	20 (71)	**17 (61)**	10 (30)	17 (52)	**11 (33)**
3	4 (14)	13 (46)	**10 (36)**	4 (12)	5 (15)	**2 (6)**
4	7 (25)	16 (57)	**10 (36)**	11 (33)	14 (42)	**7 (21)**
5	17 (61)	24 (86)	**17 (61)**	17 (52)	20 (61)	**10 (30)**
6b	13 (46)	20 (71)	**11 (39)**	19 (58)	21 (64)	**9 (27)**
7f	22 (79)	26 (93)	**13 (46)**	25 (76)	29 (88)	**10 (30)**
9V	20 (71)	25 (89)	**11 (39)**	20 (61)	25 (76)	**7 (21)**
14	26 (93)	27 (96)	**8 (29)**	31 (94)	31 (94)	**6 (18)**
18c	23 (82)	26 (93)	**14 (50)**	26 (79)	29 (88)	**13 (39)**
19A	19 (68)	22 (79)	**9 (32)**	22 (67)	23 (70)	**13 (39)**
19F	22 (79)	27 (96)	**14 (50)**	26 (79)	29 (88)	**9 (27)**
23F	18 (64)	22 (79)	**13 (46)**	17 (52)	22 (67)	**12 (36)**
≥ 8 of 12 serotypes	12 (43)	24 (86)	**7 (25)**	16 (48)	21 (64)	**6 (18)**

No significant differences were observed between controls and CKD for any of the parameters listed when α was adjusted for multiple comparisons (Bonferroni correction). N (%) shown; full definitions of “protection” and humoral response “adequacy” can be found in Methods.

Abbreviations: CKD, chronic kidney disease; Pn, pneumococcal.

### Age, Previous PPV23 Vaccination, and CMV Predict PPV23 Vaccine Response

**Figure 1.**
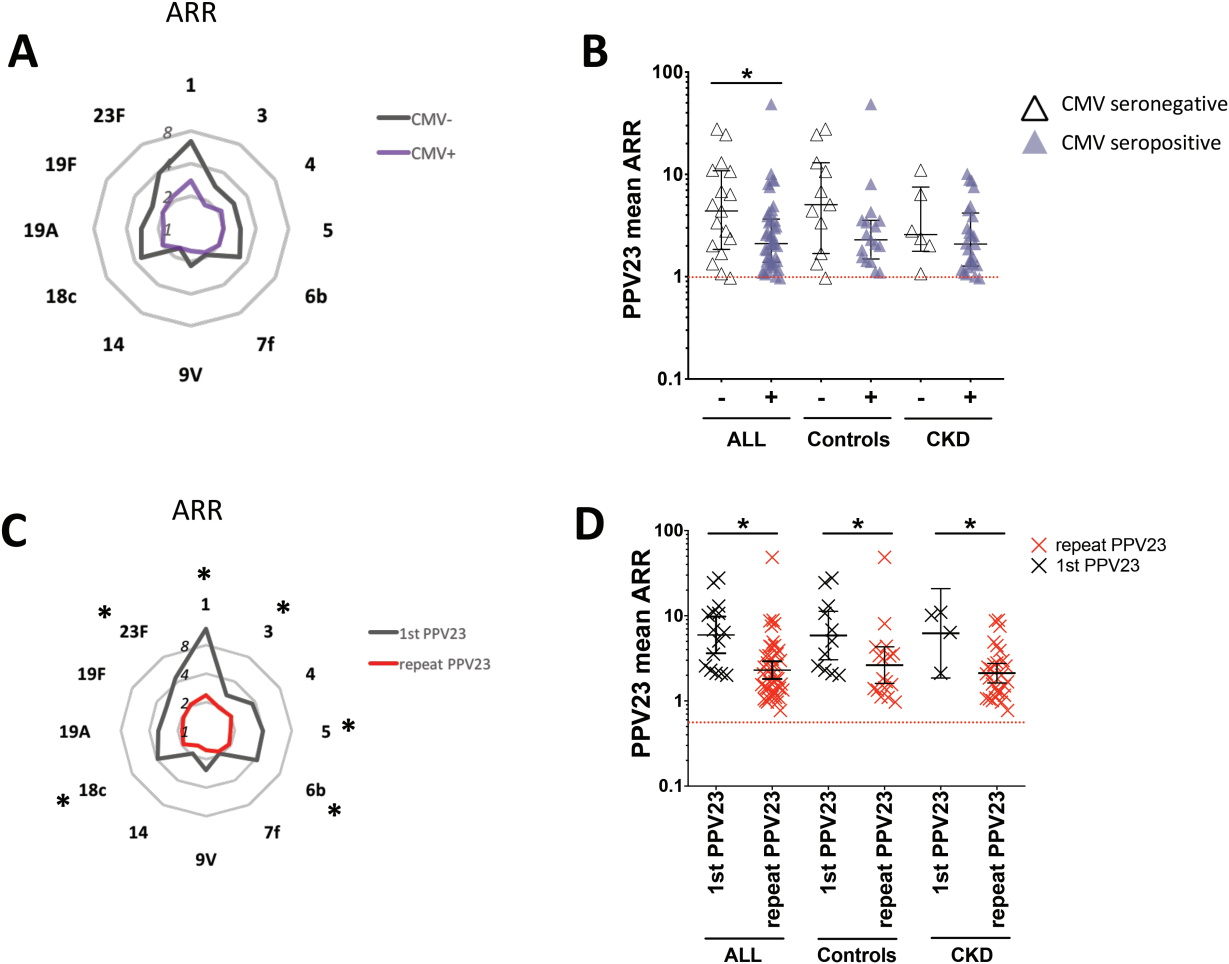
Impact of previous PPV23 vaccination and latent CMV on vaccine responses. CMV seropositivity was associated with (*A*) lower Pn serotype-specific ARRs and (*B*) significantly lower whole vaccine PPV23 ARR. CMV seropositive (+) in lilac; seronegative (-) in white. *C*, Pn serotype-specific and (*D*) whole vaccine ARR were significantly lower in PPV23 revaccinees (red symbols) than PPV23-naive individuals (gray symbols). Spider diagrams show population medians, error bars denote medians and IQR. *Denotes Mann-Whitney 2-tailed *P* < .05. Abbreviations: ARR, antibody response ratio; CMV, cytomegalovirus; IQR, interquartile range; Pn, pneumococcal; PPV23, 23-valent pneumococcal polysaccharide vaccine.

Poorer responsiveness to both vaccines was significantly associated with older age (Supplementary Figure 1), but not with CKD. Because latent CMV has previously been associated with immunosenescence [9], we then examined associations between CMV and vaccine responses. No differences were seen in whole TIV or influenza strain-specific ARRs between CMV seronegative and seropositive study participants (data not shown). However, CMV seropositive individuals demonstrated lower Pn serotype-specific ARRs and significantly poorer humoral responses to whole PPV23 when compared to their CMV seronegative counterparts (Figure 1A,B).

Given the significant differences in previous PPV23 exposure between the disease groups (Table 1), we then examined the impact of previous PPV23 vaccination on humoral responses across the whole study population. Revaccinees had significantly lower ARRs for 6 of 12 serotypes tested (Pn 1, 3, 5, 6b, 18c, and 23F) and to whole PPV23, independent of disease status (Figure 1C,D). Although revaccinees were significantly older than vaccine-naïve individuals (median, 78 years; interquartile ratio 9 vs 70, interquartile ratio 4; Mann-Whitney 2-tailed *P* < .0001), previous PPV23 vaccination remained a significant predictor of lower PPV23 ARR, independent of age, gender, smoking, and CKD status in a linear regression model (*P* = .02). Overall, specific anti-PnPS IgG titers were significantly greater at month 6 following PPV23 vaccination than at baseline (Supplementary Table 3). However, only 60% of revaccinees maintained month 6 Pn serotype-specific IgG concentrations above prevaccination levels for 8/12 serotypes tested compared with 100% of first-time PPV23 recipients (Fisher’s exact 2-tailed *P* < .01). Both CMV seropositivity and previous PPV23 vaccination significantly predicted lower PPV23 ARR in a multivariable linear regression model, independent of age, sex, smoking, or CKD status (*P* < .001 for PPV23 status, *P* = .003 for CMV status, *P* = .004 for synergistic interaction term).

### CMV Seropositivity and Associated T-cell Phenotypes, not CKD, Correlate With Vaccine Responses

**Figure 2.**
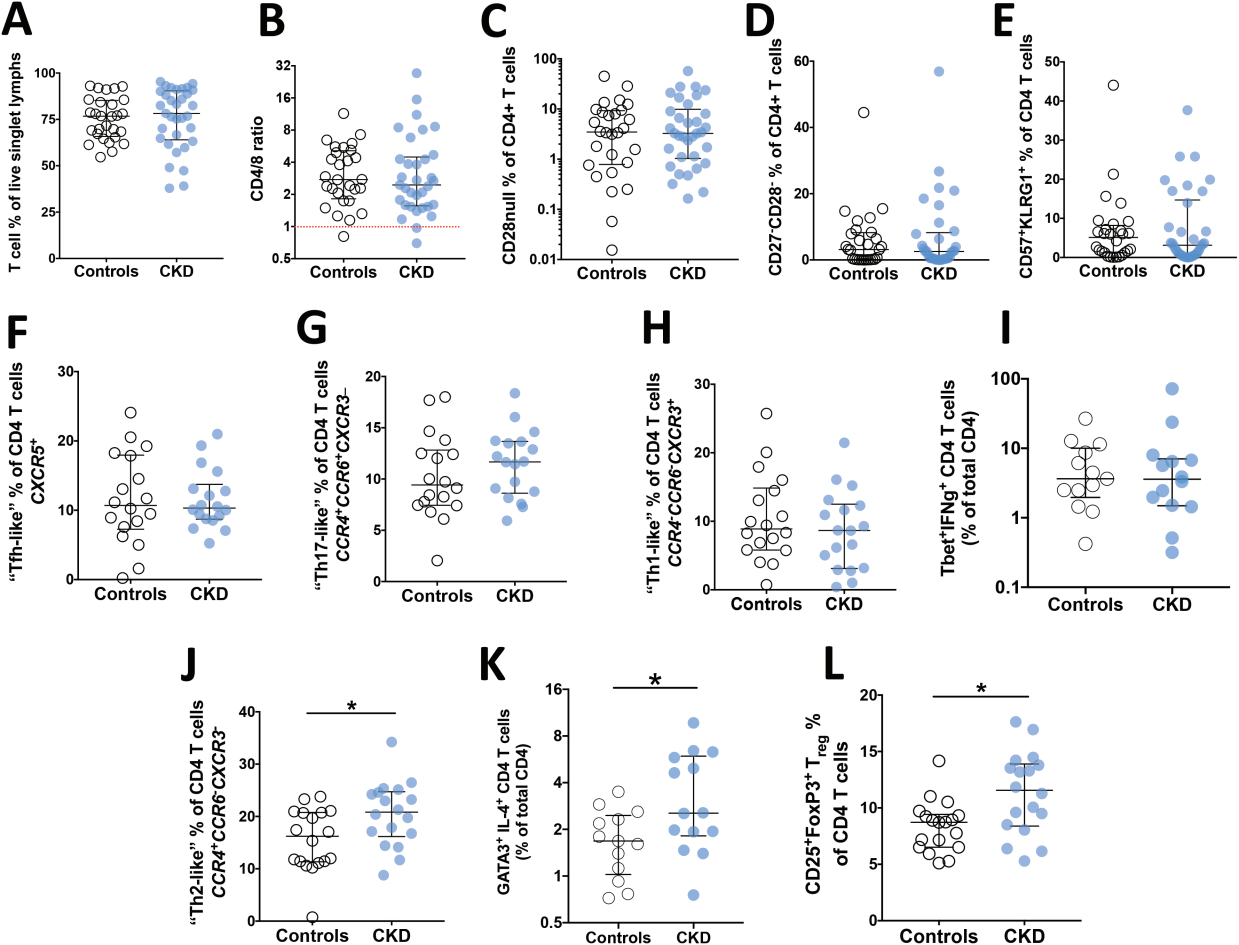
Circulating total T cells and CD4^+^ phenotypes in patients with CKD and controls. T cell % of (*A*) total lymphocytes, (*B*) CD4/8 ratio, and (*C-E*) “senescence”-associated populations are shown for patients with CKD (solid symbols) and controls (open symbols). Comparison of (*F*) T_fh_-like, (*G*) Th_17_-like, and (*H*) Th_1_ % of CD4^+^ T cells between patients with CKD and controls is shown, with the latter defined by surface chemokine receptor expression and intracellular IFN-gamma/Tbet expression after (*I*) overnight stimulation with aCD3/CD28/CD49d. Comparison of (*J*) Th_2_ % of CD4 T cells between disease groups is shown as defined by surface chemokine receptor expression and GATA3/IL-4 expression (*K*) after overnight stimulation. *L*, Comparison of CD25^+^FoxP3^+^ (T_reg_) % of CD4 T cells is shown by study group. *F –H,J*, Surface chemokine receptor staining was performed on fresh PBMCs obtained at baseline. All other phenotyping was performed using cryopreserved cells from baseline or month 6 timepoints. Error bars denote median and IQR. *Denotes Mann-Whitney 2-tailed *P* < .05. Abbreviations: CKD, chronic kidney disease; IFN, interferon; IL, interleukin; IQR, interquartile range; Th_1_, T helper 1; Th_2_, T helper 2; Th_17_, T helper 17; T_fh_, follicular T helper T; PBMC, peripheral blood mononuclear cell.

The proportion of circulating T lymphocytes, together with the CD4/8 ratio, were similar between patients with CKD and controls (Figure 2A,B; Supplementary Figure 2). Circulating proportions of CD45RA- and CCR7-expressing naïve/memory and CD27^-^/28^-^ or CD57^+^KLRG1^+^ terminally differentiated CD4^+^ and CD8^+^ T cells were also similar between the groups (Figure 2*C-E* and Supplementary Figures 3,4), as were populations of “T_fh_-like” (CXCR5^+^), “T_h17_-like” (CCR4^+^CCR6^+^CXCR3^-^), and T_h1_ CD4^+^ T cells (CCR4^-^CCR6^-^CXCR3^+^ or IFNg^+^Tbet^+^) (Figure 2*F-I*; Supplementary Figure 5). However, T_h2_ (CCR4^+^CCR6^-^CXCR3^-^ or GATA3^+^IL-4^+^) and regulatory T cell (T_reg_) (CD25^high^FoxP3^+^) CD4^+^ T cells were significantly expanded in patients with CKD compared with controls (Figure 2*J-L*; Supplementary Figure 6).

**Figure 3.**
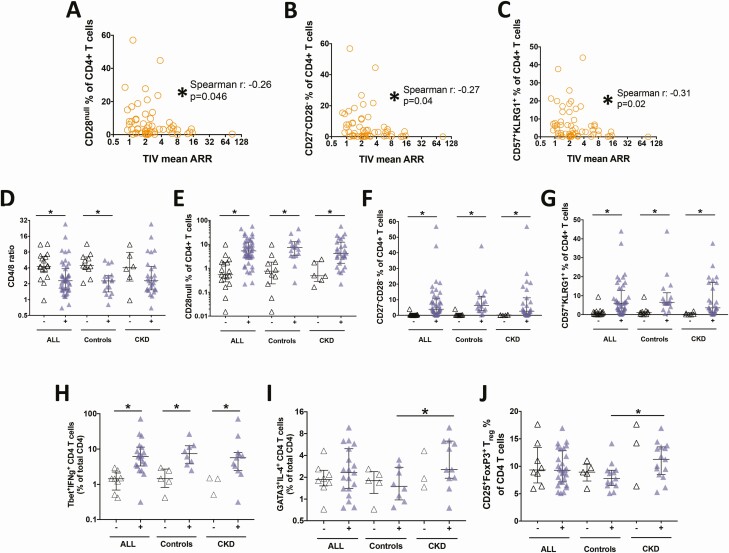
Impact of latent CMV infection on TIV response and circulating T-cell phenotypes. (*A-C*) Lower TIV mean ARRs were significantly associated with 3 CD4^+^ “senescence” and CMV-associated phenotypes. Latent CMV infection was associated with (*D*) a lower CD4/8 ratio and (*E-G*) expansions of “senescence”-associated T-cell populations. CMV seropositive (+): solid symbols; seronegative (-): open symbols. Although CMV seropositivity was associated with (*H*) greater circulating Th_1_ populations, it did not appear to affect (*I,J*) Th_2_ or T_reg_ populations. *Denotes Mann-Whitney 2-tailed *P* < .05 unless stated. Abbreviations: ARR, antibody response ratio; CMV, cytomegalovirus; Th_1_, T helper 1; Th_2_, T helper 2; TIV, trivalent inactivated influenza; T_reg_, regulatory T cell.

As expected with T-independent antigens, we did not observe any significant associations between circulating T-cell populations and PPV23 ARR. TIV ARR was not related to circulating T_h2_ or T_reg_ populations, but a significant inverse association was seen with proportions of terminally differentiated CD4^+^ T cells (Figure 3*A-C*). Both CKD and CMV have previously been associated with expansions of such CD4^+^ T-cell subsets, most notably CD4^+^CD28^null^ cells [18, 19]. CMV seropositivity, not CKD status, was the main correlate of the CD4/8 ratio and the size of CD4^+^CD28^null^ and other terminally differentiated T-cell populations (Figure 3*D-G*; Supplementary Figure 4). CMV seropositivity was not associated with the size of T_h2_ or T_reg_ populations (Figure 3*I,J*).

**Figure 4.**
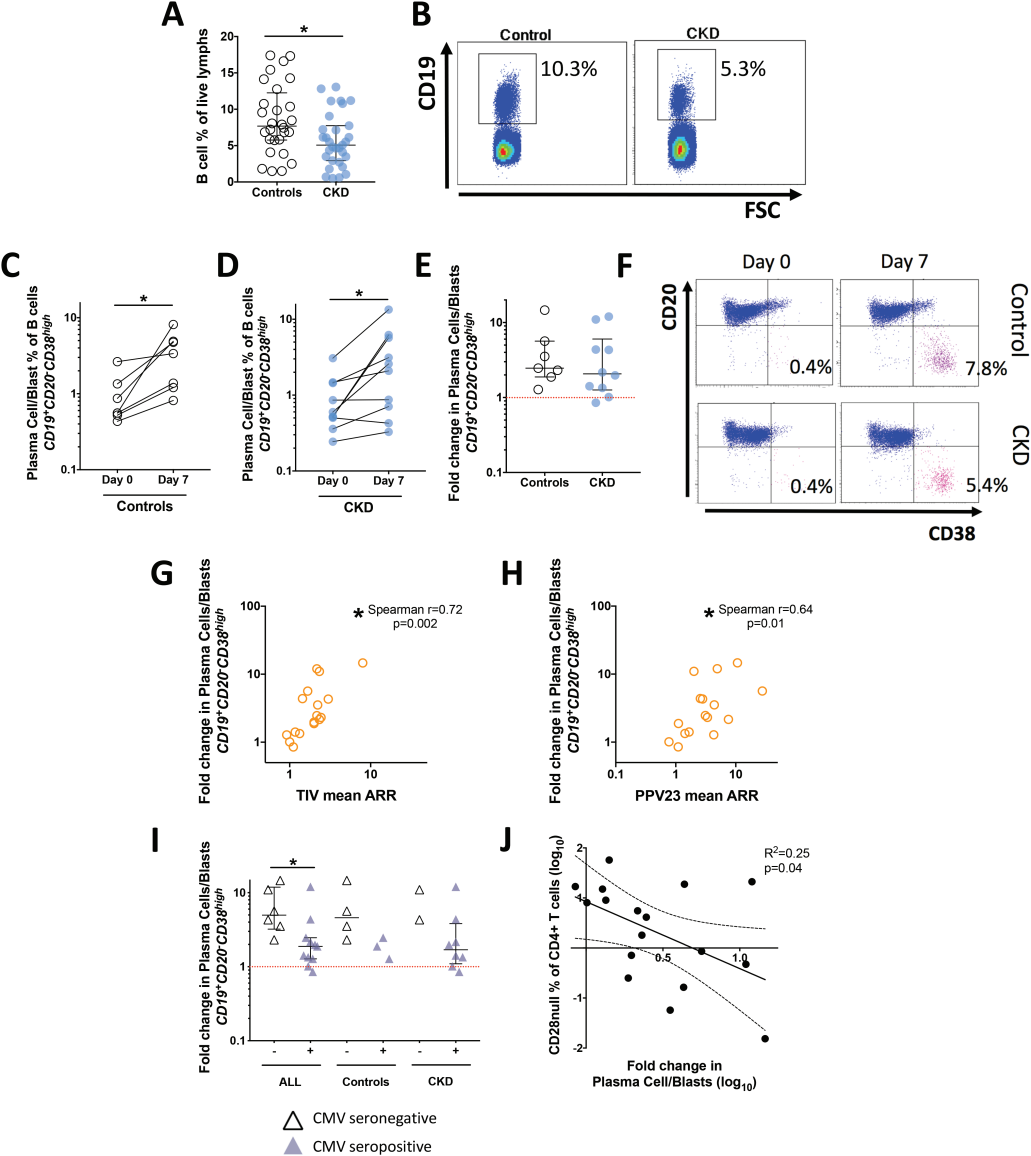
Effects of CKD and latent CMV on plasma cell/blast generation following vaccination. B cell % of total B cells are shown for patients with CKD (solid circle symbols) and controls (open circle symbols) in (*A*) with representative gating and (*B*) density plots; baseline/month 6 cryopreserved PBMCs. *C,D* The absolute change and (*E*) fold change (day 7/baseline, dotted line denotes no change) in circulating PC/Bs (defined as CD19^+^CD29^+^CD38^high^ % of total B cells) are shown together with representative scatter plots at (*F*) baseline and day 7. (*G,H*) PB/C fold change significantly correlated with vaccine response. CMV seropositivity was associated with (*I*) significantly lower fold change in circulating plasma cells/blasts and (*J*) expansions of CD28^null^ CD4^+^ T cells (phenotype associated with latent CMV infection) were significantly associated with smaller fold change of circulating PC/Bs at day 7 (Pearson’s statistics shown, performed on normalized data). *Denotes Mann-Whitney 2-tailed *P* < .05 unless stated. Abbreviations: CKD, chronic kidney disease; CMV, cytomegalovirus; PC/B, plasma cell/blast; PBMC, peripheral blood mononuclear cell.

In keeping with previous data [20, 21], patients with CKD had a significantly lower proportion of circulating B cells (relative to total circulating lymphocytes) than controls (Figure 4*A*). Despite this, patients with CKD were able to expand circulating PC/B from baseline to day 7 postvaccination to the same degree as controls (Figure 4*C-F*), reflecting the similar humoral vaccine responses observed. The fold change in circulating PC/B proportion of total B cells (day 7/day 0) significantly correlated with both PPV23 and TIV ARR (Figure 4*G,H*). CMV seropositivity was associated with smaller expansions of PC/Bs at day 7 postvaccination (Figure 4*I*), which were also, in turn, associated with larger proportions of CD4^+^CD28^null^ T cells (Figure 4*J*).

## DISCUSSION

This prospective study in older adults with and without CKD examined humoral responses to simultaneous vaccination with TIV and PPV23 alongside cross-sectional profiling of circulating lymphocyte subsets. Vaccine responses were modest overall and similar between disease groups, consistent with previous work showing that functional antibody responses can be elicited in older patients with CKD [6]. In keeping with this, both groups also demonstrated similar fold-increases in circulating PC/Bs after vaccination. This shows that B-cell responsiveness is maintained in CKD, despite the lower proportion of circulating B cells detected compared with controls. Older age, previous PPV23 vaccination, and CMV seropositivity were predictors of poor vaccine response, whereas CKD was not. Previous studies investigating TIV and PPV23 responsiveness in adults with nondialysis CKD have reported variable results [22–24], which may reflect heterogeneity of populations studied. Indeed, many such studies included patients on dialysis, which is known to exert independent immunomodulatory effects [25, 26], and were less controlled for age and frailty [27]. Our findings are paralleled by a recent large epidemiological study that found responses to influenza and pneumococcal vaccines were poor in older patients with diabetes and not affected by CKD, unless patients required dialysis [28].

Surprisingly, patients with CKD had no reduction in proportions of circulating total T cells or other phenotypic differences previously described [29–31]. However, CMV seropositivity strongly correlated with a higher frequency of T_h1_ cells and terminally differentiated CD4^+^CD28^null^ T cells in both controls and patients with CKD. These associations with CMV are known [32], but we also observed that expansions of CD4^+^CD28^null^ T cells were negatively correlated with vaccine response.

Primary CMV infections typically occur in childhood and are clinically nonspecific or asymptomatic, making the timing of CMV acquisition difficult [33]. Following infection, the virus establishes a persistent state termed “latent,” characterized by periodic episodes of subclinical reactivation triggered by biological stressors [18]. Such reactivations are transient and rarely detectable in blood [34]. Although we did not evaluate CMV viremia in our study population, reports suggest that frequent episodes of subclinical CMV reactivation are associated with higher levels of CMV-specific IgG [34]. In our study, prevalence of CMV seropositivity and titers of CMV-specific IgG were similar in controls and patients with CKD, suggesting a similar CMV burden in both study groups. This is likely to explain the differences in our results from other studies, which do not control for the immunomodulating effects of CMV.

Published data on whether CMV seropositivity correlates with vaccine responses are inconsistent [35]. However, several studies suggest that the magnitude of the immune response to latent CMV infection is more important than simply CMV seropositivity. In vitro studies suggest that inflation of the CMV memory response, including expansions of potentially cytotoxic, but poorly replicative CD4^+^CD28^null^ T cells is associated with a progressive decline in the overall functional capacity of T cells [34, 36]. We identified negative correlations between CD4^+^CD28^null^ T cells and humoral responses to a tetanus-diphtheria vaccine (TIV), together with day 7 PC/B expansions. CMV seropositivity was also associated with poorer responses to the TIV (PPV23) in the study population as a whole. CMV-associated impairment of B-cell responses has previously been reported with regard to tetanus-diphtheria vaccines [10, 11], but not for TIVs. There are several potential mechanisms by which CMV could impact TIV antigen responses and further studies are needed to investigate these nonexhaustive options. For example, T_fh_ cells are known to play a significant role in generating long-lived memory B-cell responses to pneumococcal polysaccharides [37] and it is possible that latent CMV may result in T_fh_ dysfunction. In addition, CMV infects cells of the monocyte lineage [18], and secretion of B-cell survival factors by macrophages is known to promote TIV class switch recombination [38]. As such, the influence of CMV in TIV responses may be through dysfunction of mechanisms supporting plasma cell differentiation and/or survival. Our findings suggest that latent CMV infection could play a greater role in modulating immune responses to vaccines in older adults, with and without chronic disease, than is currently appreciated. A recent proof-of-concept study in patients with antineutrophil cytoplasmic antibody–associated vasculitis showed improved vaccine responses following suppression of CMV reactivation [34]. The interesting associations we observed between CMV serostatus and vaccine responses were, however, assessed post hoc as exploratory analyses and therefore require confirmation in future studies.

Responses to PPV23 were lower in those who had previously received PPV23, despite a median 10-year interval between immunizations. This is in keeping with immune hyporesponsiveness to repeat plain polysaccharide vaccination [39] and consistent with previous studies showing only marginal reductions in morbidity and mortality with PPV23 vaccination in older adults [40]. Interestingly, both CMV seropositivity and previous PPV23 vaccination were significant predictors of PPV23 mean ARR, independent of age, sex, smoking, and CKD status. Although we controlled for multiple confounders that could explain the reduced responsiveness with repeat PPV23 immunization, there may be other unmeasured contributory factors. As such, further studies are needed to determine the impact of repeat PPV23 vaccination and evaluate whether conjugate pneumococcal vaccines may be advantageous in high-risk individuals.

Limitations of the study include cohort size and immunization over 3 influenza seasons, which may have affected our ability to detect subtle differences in humoral responses. However, patients with CKD were well-controlled for age and sex. The majority of the CKD cohort had moderate/severe renal impairment with significant proteinuria and clinical features representative of the wider CKD population (Table 1). Although several potential immunomodulating factors (including dialysis therapy and autoimmune disease) were excluded through strict patient selection, we were not able to control for all potential confounders in this study. Our study has only investigated lymphocyte function through antibody responses and cytokine production. Thus, it is possible that defects in antigen presentation and innate immune system activation could be present in CKD-associated immune dysfunction and may be more important than lymphocyte function. The evaluation of the impact of CMV serostatus and prior PPV23 vaccination on vaccine responses constitute exploratory analyses and our findings therefore require independent confirmation in further studies that should also include investigation of potential mechanisms, which we did not perform as part of this work.

This study and our previous work [6] suggest that, to understand the etiology of increased infection risk in CKD, we need to examine how the adaptive and innate immune systems interact to control infection. We suggest that future studies of immune function in this field be controlled for the presence of latent CMV infection because of its large effect on shaping immune phenotypes and that the effect of subclinical episodes of CMV reactivation on immune responses is investigated. Finally, our observation of significant and long-lasting polysaccharide hyporesponsiveness in older adults, with and without chronic disease, suggests that further studies are required to examine the immunological and clinical impact of repeated plain polysaccharide versus conjugate antipneumococcal vaccines in this vulnerable population.

## Supplementary Material

ciab078_suppl_Supplementary_Data

## Notes

***Acknowledgments.*** The authors acknowledge the support of the staff in the renal outpatient department, the National Institute for Health Research/Wellcome Trust Clinical Research Facility and the research and development department at University Hospitals Birmingham NHS Foundation Trust, together with all study participants.

***Funding.*** This work was supported by a Wellcome Trust Joint Basic and Clinical PhD Studentship (105479/Z/14/Z; www.wellcome.ac.uk) awarded to N. W.

***Potential conflicts of interest.*** A. C. reports personal fees form Sanofi, outside the submitted work. The other authors declare no conflict of interests. All authors have submitted the ICMJE Form for Disclosure of Potential Conflicts of Interest. Conflicts that the editors consider relevant to the content of the manuscript have been disclosed.

## References

[1] Stevens PE, O’Donoghue DJ, de Lusignan S, et al. Chronic kidney disease management in the United Kingdom: NEOERICA project results. Kidney Int 2007; 72:92–9. 17440495 10.1038/sj.ki.5002273

[2] Steenkamp R, Rao A, Fraser S. UK Renal Registry 18th Annual Report (December 2015) Chapter 5: survival and causes of death in UK adult patients on renal replacement therapy in 2014: national and centre-specific analyses. Nephron 2016; 132(Suppl 1):111–44. 27115403 10.1159/000444819

[3] James MT, Laupland KB, Tonelli M, Manns BJ, Culleton BF, Hemmelgarn BR; Alberta Kidney Disease Network. Risk of bloodstream infection in patients with chronic kidney disease not treated with dialysis. Arch Intern Med 2008; 168:2333–9. 19029498 10.1001/archinte.168.21.2333

[4] Fleming SJ, Moran DM, Cooksley WG, Faoagali JL. Poor response to a recombinant hepatitis B vaccine in dialysis patients. J Infect 1991; 22:251–7. 1830073 10.1016/s0163-4453(05)80007-6

[5] Peces R, Laurés AS. Persistence of immunologic memory in long-term hemodialysis patients and healthcare workers given hepatitis B vaccine: role of a booster dose on antibody response. Nephron 2001; 89:172–6. 11549899 10.1159/000046064

[6] Wall NA, Dominguez-Medina CC, Faustini SE, et al. Humoral immunity to memory antigens and pathogens is maintained in patients with chronic kidney disease. PLoS One 2018; 13:e0195730. 29659606 10.1371/journal.pone.0195730 PMC5901993

[7] Crooke SN, Ovsyannikova IG, Poland GA, Kennedy RB. Immunosenescence and human vaccine immune responses. Immun Ageing 2019; 16:25. 31528180 10.1186/s12979-019-0164-9 PMC6743147

[8] Russell FM, Carapetis JR, Balloch A, et al. Hyporesponsiveness to re-challenge dose following pneumococcal polysaccharide vaccine at 12 months of age, a randomized controlled trial. Vaccine 2010; 28:3341–9. 20206670 10.1016/j.vaccine.2010.02.087 PMC2854305

[9] Trzonkowski P, Myśliwska J, Szmit E, et al. Association between cytomegalovirus infection, enhanced proinflammatory response and low level of anti-hemagglutinins during the anti-influenza vaccination–an impact of immunosenescence. Vaccine 2003; 21:3826–36. 12922116 10.1016/s0264-410x(03)00309-8

[10] Derhovanessian E, Theeten H, Hähnel K, Van Damme P, Cools N, Pawelec G. Cytomegalovirus-associated accumulation of late-differentiated CD4 T-cells correlates with poor humoral response to influenza vaccination. Vaccine 2013; 31:685–90. 23196209 10.1016/j.vaccine.2012.11.041

[11] Frasca D, Diaz A, Romero M, Landin AM, Blomberg BB. Cytomegalovirus (CMV) seropositivity decreases B cell responses to the influenza vaccine. Vaccine 2015; 33:1433–9. 25659271 10.1016/j.vaccine.2015.01.071 PMC4352374

[12] Chanouzas D, Sagmeister M, Faustini S, et al. Subclinical reactivation of cytomegalovirus drives CD4+CD28null T-cell expansion and impaired immune response to pneumococcal vaccination in antineutrophil cytoplasmic antibody-associated vasculitis. J Infect Dis 2019; 219:234–44. 30102389 10.1093/infdis/jiy493 PMC6306020

[13] Vaccines against influenza WHO position paper – November 2012. Wkly Epidemiol Rec 2012; 87:461–76. Available at: http://www.who.int/wer/2012/wer8747.pdf?ua=1. Accessed March 18, 2021. 23210147

[14] Harvey R, Nicolson C, Johnson RE, et al. Improved haemagglutinin antigen content in H5N1 candidate vaccine viruses with chimeric haemagglutinin molecules. Vaccine 2010; 28:8008–14. 20934460 10.1016/j.vaccine.2010.09.006

[15] Whitelegg AM, Birtwistle J, Richter A, et al. Measurement of antibodies to pneumococcal, meningococcal and haemophilus polysaccharides, and tetanus and diphtheria toxoids using a 19-plexed assay. J Immunol Methods 2012; 377:37–46. 22293629 10.1016/j.jim.2012.01.007

[16] WHO Publication. pneumococcal vaccines WHO position paper - 2012 - recommendations. Vaccine 2012; 30:4717–8. 22621828 10.1016/j.vaccine.2012.04.093

[17] Wall NA, Chue CD, Edwards NC, et al. Cytomegalovirus seropositivity is associated with increased arterial stiffness in patients with chronic kidney disease. PLoS One 2013; 8:e55686. 23451030 10.1371/journal.pone.0055686 PMC3581505

[18] Koch S, Larbi A, Ozcelik D, et al. Cytomegalovirus infection: a driving force in human T cell immunosenescence. Ann N Y Acad Sci 2007; 1114:23–35. 17986574 10.1196/annals.1396.043

[19] Betjes MG, de Wit EE, Weimar W, Litjens NH. Circulating pro-inflammatory CD4posCD28null T cells are independently associated with cardiovascular disease in ESRD patients. Nephrol Dial Transplant 2010; 25:3640–6. 20400452 10.1093/ndt/gfq203

[20] Xiang FF, Zhu JM, Cao XS, et al. Lymphocyte depletion and subset alteration correlate to renal function in chronic kidney disease patients. Ren Fail 2016; 38:7–14. 26539739 10.3109/0886022X.2015.1106871

[21] Fernández-Fresnedo G, Ramos MA, González-Pardo MC, de Francisco AL, López-Hoyos M, Arias M. B lymphopenia in uremia is related to an accelerated in vitro apoptosis and dysregulation of Bcl-2. Nephrol Dial Transplant 2000; 15:502–10. 10727545 10.1093/ndt/15.4.502

[22] Kausz AT, Gilbertson DT. Overview of vaccination in chronic kidney disease. Adv Chronic Kidney Dis 2006; 13:209–14. 16815227 10.1053/j.ackd.2006.04.007

[23] Cavdar C, Sayan M, Sifil A, et al. The comparison of antibody response to influenza vaccination in continuous ambulatory peritoneal dialysis, hemodialysis and renal transplantation patients. Scand J Urol Nephrol 2003; 37:71–6. 12745749 10.1080/00365590310008749

[24] Vogtländer NP, Brown A, Valentijn RM, Rimmelzwaan GF, Osterhaus AD. Impaired response rates, but satisfying protection rates to influenza vaccination in dialysis patients. Vaccine 2004; 22:2199–201. 15149777 10.1016/j.vaccine.2003.11.046

[25] Mai K, Boldt A, Hau HM, et al. Immunological alterations due to hemodialysis might interfere with early complications in renal transplantation. Anal Cell Pathol (Amst) 2019; 2019:8389765. 31019876 10.1155/2019/8389765 PMC6452532

[26] Sardenberg C, Suassuna P, Andreoli MC, et al. Effects of uraemia and dialysis modality on polymorphonuclear cell apoptosis and function. Nephrol Dial Transplant 2006; 21:160–5. 16155068 10.1093/ndt/gfi095

[27] McElhaney JE, Andrew MK, McNeil SA. Estimating influenza vaccine effectiveness: evolution of methods to better understand effects of confounding in older adults. Vaccine 2017; 35:6269–74. 29032898 10.1016/j.vaccine.2017.09.084

[28] McDonald HI, Thomas SL, Millett ERC, Quint J, Nitsch D. Do influenza and pneumococcal vaccines prevent community-acquired respiratory infections among older people with diabetes and does this vary by chronic kidney disease? A cohort study using electronic health records. BMJ Open Diabetes Res Care 2017; 5:e000332. 10.1136/bmjdrc-2016-000332 PMC5387965 28461899

[29] Girndt M, Sester M, Sester U, Kaul H, Köhler H. Molecular aspects of T- and B-cell function in uremia. Kidney Int Suppl 2001; 78:S206–11. 11169012 10.1046/j.1523-1755.2001.59780206.x

[30] Yoon JW, Gollapudi S, Pahl MV, Vaziri ND. Naïve and central memory T-cell lymphopenia in end-stage renal disease. Kidney Int 2006; 70:371–6. 16738532 10.1038/sj.ki.5001550

[31] Litjens NH, van Druningen CJ, Betjes MG. Progressive loss of renal function is associated with activation and depletion of naive T lymphocytes. Clin Immunol 2006; 118:83–91. 16257266 10.1016/j.clim.2005.09.007

[32] Morgan MD, Pachnio A, Begum J, et al. CD4+CD28- T cell expansion in granulomatosis with polyangiitis (Wegener’s) is driven by latent cytomegalovirus infection and is associated with an increased risk of infection and mortality. Arthritis Rheum 2011; 63:2127–37. 21437878 10.1002/art.30366

[33] Cannon MJ, Schmid DS, Hyde TB. Review of cytomegalovirus seroprevalence and demographic characteristics associated with infection. Rev Med Virol 2010; 20:202–13. 20564615 10.1002/rmv.655

[34] Chanouzas D, Sagmeister M, Faustini S, Nightingale P, Richter A, Ferro CJ, Morgan MD, Moss P, Harper L. Subclinical reactivation of cytomegalovirus drives CD4+CD28null T-cell expansion and impaired immune response to pneumococcal vaccination in antineutrophil cytoplasmic antibody-associated vasculitis. J Infect Dis 2019; 219:234–44. 30102389 10.1093/infdis/jiy493 PMC6306020

[35] van den Berg SPH, Warmink K, Borghans JAM, Knol MJ, van Baarle D. Effect of latent cytomegalovirus infection on the antibody response to influenza vaccination: a systematic review and meta-analysis. Med Microbiol Immunol 2019; 208:305–21. 30949763 10.1007/s00430-019-00602-z PMC6647367

[36] Derhovanessian E, Maier AB, Hähnel K, McElhaney JE, Slagboom EP, Pawelec G. Latent infection with cytomegalovirus is associated with poor memory CD4 responses to influenza A core proteins in the elderly. J Immunol (Baltimore, Md: 1950) 2014; 193:3624–31. 10.4049/jimmunol.1303361 25187662

[37] Jha V, Janoff EN. Complementary role of CD4+ T cells in response to pneumococcal polysaccharide vaccines in Humans. Vaccines (Basel) 2019; 7:18. 10.3390/vaccines7010018 PMC6466080 30754689

[38] Xu W, Banchereau J. The antigen presenting cells instruct plasma cell differentiation. Front Immunol 2014; 4:504. 24432021 10.3389/fimmu.2013.00504 PMC3880943

[39] Poolman J, Borrow R. Hyporesponsiveness and its clinical implications after vaccination with polysaccharide or glycoconjugate vaccines. Expert Rev Vaccines 2011; 10:307–22. 21434799 10.1586/erv.11.8

[40] Moberley S, Holden J, Tatham DP, Andrews RM. Vaccines for preventing pneumococcal infection in adults. Cochrane Database System Rev 2013; Cd000422. 23440780 10.1002/14651858.CD000422.pub3 PMC7045867

