# Supplementary material for: Latent Cytomegalovirus Infection and Previous Capsular Polysaccharide Vaccination Predict Poor Vaccine Responses in Older Adults, Independent of Chronic Kidney Disease
Source: Clin Infect Dis. 2021 Mar 16;73(4):e880–9. doi: 10.1093/cid/ciab078 (PMC8366832; doi:10.1093/cid/ciab078)

Supplementary data

Supplementary Table 1

Antibodies used in flow cytometry experiments. Abbreviations: CD – cluster of differentiation, BV – Brilliant Violet, PE – phycoerythrin, APC - allophycocyanine, Cy – Cyanine, FITC – fluorescein isothiocyanate, PerCP – peridinin chlorophyll protein, BB – Brilliant Blue, IFNg – interferon gamma, IL– interleukin, Ig – immunoglobulin.

Antibody target	Manufacturer	Clone	Fluorochrome
CD3	eBioscience	OKT3	-
CD28	eBioscience	CD28.2	-
CD49d	eBioscience	9F10	-
CD3	BD Biosciences	UCTH1	Alexa Fluor 700
CD4	BD Biosciences	SK3	BV711
CD4	BD Biosciences	RPA-T4	BV605
CD8	BD Biosciences	RPA-T8	BV510
CD45RA	BD Biosciences	HI100	BV421
CCR7/CD197	BD Biosciences	150503	PE-CF594
CD25	BD Biosciences	M-A251	APC
CD27	BD Biosciences	M-T271	PE-Cy7
CD27	BD Biosciences	L128	BV650
CD28	BD Biosciences	CD28.2	APC
CD57	Biolegend	HNK-1	PE
KLRG1	Biolegend	SA231A2	FITC
CCR4/CD194	Biolegend	L291H4	PE-Cy7
CXCR5/CD185	Biolegend	J252D4	APC
CXCR3/CD183	Biolegend	G025H7	FITC
CCR6/CD196	Biolegend	G034E3	PerCP-Cy5.5
FoxP3	eBioscience	PCH101	PE
GATA3	BD Biosciences	L50-823	BB700
Tbet	Biolegend	4B10	PE
IFNg	Biolegend	B27	PE-Cy7
IL-4	Biolegend	8D4-8	APC
CD19	BD Biosciences	HIB19	BB515
CD20	BD Biosciences	2H7	Alexa Fluor 700
IgD	BD Biosciences	IA6-2	PE-CF594
CD38	Biolegend	HIT2	BV510

Supplementary Table 2

Strain-specific influenza vaccine humoral responses. Serotype-specific HAI titres at baseline (day 0), peak (day 28) and month 6 are compared between controls and patients with CKD. No significant differences were observed between controls and CKD for any of the parameters listed when α was adjusted for multiple comparisons (Bonferroni correction). ARR: geometric mean of antibody response ratio, AMR: geometric mean of antibody maintenance ratio. Data was log transformed for statistical analysis. * - p<0.05 2-tailed paired t test: comparison of Day 0 and Day 28 titres within controls/patients with CKD; ~ - p<0.05 2-tailed paired t test: comparison of Day 28 and Month 6 titres within controls/patients with CKD; # - p<0.05 2-tailed paired t test: comparison of Month 6 and Day 0 titres within controls/patients with CKD.

**Influenza strain**	**Timepoint**	**Controls**		**CKD**	
		n	GMT (95% CI)	n	GMT (95% CI)
**A/H1N1**	Day 0	28	21.7 (14.8-31.9)	33	19.6 (12.8-29.8)
	Day 28	28	56.8 (39.0-82.6)*	33	52.0 (31.8-85.0)*
	Month 6	27	37.2 (25.4-54.5)~#	30	24.6 (14.6-41.4)~
	**ARR**		**2.6 (1.8-3.8)**		**2.7 (1.7-4.1)**
	**AMR**		**0.65 (0.52-0.80)**		**0.58 (0.43-0.77)**
**A/H3N2**	Day 0	28	50.0 (25.6-97.4)	33	51.7 (31.9-83.7)
	Day 28	28	111.8 (66.3-188.3)*	33	104.6 (64.2-170.3)*
	Month 6	27	81.9 (46.0-146.0)~#	30	77.3 (47.3-126.3)~#
	**ARR**		**2.2 (1.6-3.2)**		**2.0 (1.4-2.9)**
	**AMR**		**0.74 (0.58-0.95)**		**0.71 (0.56-0.91)**
**B**	Day 0	28	9.6 (6.3-14.4)	33	7.7 (6.0-10.0)
	Day 28	28	15.8 (10.1-24.6)*	33	11.9 (8.2-17.1)*
	Month 6	27	11.7 (7.6-18.1)~#	30	9.8 (6.8-14.1)~#
	**ARR**		**1.6 (1.2-2.2)**		**1.5 (1.2-2.0)**
	**AMR**		**0.75 (0.62-0.91)**		**0.80 (0.68-0.95)**

Supplementary Table 3

Serotype-specific anti-Pneumococcal IgG responses. Serotype-specific anti-Pn IgG titres are shown at baseline (day 0), peak (day 28) and month 6 post-vaccination with PPV23, compared between controls and patients with CKD. Geometric mean concentrations (GMC) and 95% CI shown with unpaired t-test 2-tailed p value for log-transformed data. No significant differences were observed between controls and CKD for any of the parameters listed when α was adjusted for multiple comparisons (Bonferroni correction). ARR: antibody response ratio, AMR: antibody maintenance ratio. *p<0.05 comparison of Day 0 and Day 28 titres; ~p<0.05 comparison of Day 28 and Month 6 titre; $^{\#}$p<0.05 comparison of Month 6 and Day 0 titre.

		Controls		CKD	
		n	GMC (95% CI)	n	GMC (95% CI)
**Pn1**	Day 0	28	0.29 (0.14-0.59)	33	0.15 (0.08-0.28)
	Day 28	28	1.30*(0.63-2.65)	33	0.46*(0.24-0.90)
	Month 6	27	1.13~$^{\#}$(0.53-2.42)	30	0.35~$^{\#}$(0.17-0.74)
	**ARR**		**4.4 (2.8-7.1)**		**3.0 (1.9-4.7)**
	**AMR**		**0.83 (0.72-0.96)**		**0.76 (0.67-0.86)**
**Pn3**	Day 0	28	0.14 (0.08-0.24)	33	0.08 (0.05-0.13)
	Day 28	28	0.29*(0.15-0.54)	33	0.15*(0.09-0.26)
	Month 6	27	0.26$^{\#}$(0.14-0.48)	30	0.11~$^{\#}$(0.06-0.19)
	**ARR**		**2.3 (1.6-3.2)**		**1.9 (1.5-2.4)**
	**AMR**		**0.98 (0.79-1.21)**		**0.75 (0.65-0.86)**
**Pn4**	Day 0	28	0.15 (0.08-0.27)	33	0.12 (0.07-0.21)
	Day 28	28	0.39*(0.22-0.67)	33	0.24*(0.14-0.41)
	Month 6	27	0.35~$^{\#}$(0.19-0.62)	30	0.19~$^{\#}$(0.11-0.35)
	**ARR**		**2.7 (1.9-4.0)**		**2.0 (1.5-2.7)**
	**AMR**		**0.84 (0.74-0.95)**		**0.78 (0.69-0.89)**
**Pn5**	Day 0	28	0.63 (0.37-1.07)	33	0.48 (0.28-0.80)
	Day 28	28	1.70*(0.97-2.98)	33	0.93*(0.51-1.67)
	Month 6	27	1.83$^{\#}$(1.10-3.04)	30	0.69~$^{\#}$(0.38-1.25)
	**ARR**		**2.5 (1.7-3.7)**		**2.0 (1.4-2.8)**
	**AMR**		**0.95 (0.83-1.08)**		**0.83 (0.72-0.96)**
**Pn6b**	Day 0	28	0.29 (0.15-0.57)	33	0.44 (0.24-0.79)
	Day 28	28	0.86*(0.42-1.76)	33	0.77*(0.42-1.42)
	Month 6	27	0.69~$^{\#}$(0.32-1.46)	30	0.60~$^{\#}$(0.30-1.19)
	**ARR**		**3.0 (1.8-5.0)**		**1.9 (1.3-2.6)**
	**AMR**		**0.81 (0.73-0.91)**		**0.81 (0.72-0.93)**
**Pn7f**	Day 0	28	1.14 (0.69-1.87)	33	0.84 (0.54-1.30)
	Day 28	28	2.20*(1.41-3.44)	33	1.42*(0.92-2.19)
	Month 6	27	1.88~$^{\#}$(1.18-3.00)	30	1.30~$^{\#}$(0.80-2.12)
	**ARR**		**2.0 (1.5-2.7)**		**1.7 (1.3-2.3)**
	**AMR**		**0.88 (0.80-0.98)**		**0.87 (0.77-0.98)**
**Pn9V**	Day 0	28	0.61 (0.40-0.93)	33	0.51 (0.34-0.78)
	Day 28	28	1.19*(0.76-1.86)	33	0.82*(0.52-1.29)
	Month 6	27	1.15$^{\#}$(0.73-1.80)	30	0.72$^{\#}$(0.45-1.14)
	**ARR**		**2.0 (1.4-2.8)**		**1.6 (1.2-2.3)**
	**AMR**		**0.95 (0.86-1.04)**		**0.88 (0.75-1.02)**
**Pn14**	Day 0	28	2.22 (1.33-3.72)	33	2.40 (1.59-3.62)
	Day 28	28	3.92*(2.60-5.90)	33	3.33*(2.19-5.06)
	Month 6	27	3.85$^{\#}$(2.51-5.91)	30	3.00$^{\#}$(1.92-4.68)
	**ARR**		**1.8 (1.3-2.5)**		**1.5 (1.2-1.8)**
	**AMR**		**0.95 (0.88-1.04)**		**0.92 (0.81-1.05)**
**Pn18c**	Day 0	28	1.10 (0.66-1.84)	33	1.49 (0.89-2.51)
	Day 28	28	2.84*(1.71-4.72)	33	2.86*(1.70-4.80)
	Month 6	27	2.52$^{\#}$(1.46-4.36)	30	2.65$^{\#}$(1.47-4.78)
	**ARR**		**2.8 (1.9-4.2)**		**1.9 (1.5-2.5)**
	**AMR**		**0.90 (0.80-1.00)**		**0.94 (0.87-1.03)**
**Pn19A**	Day 0	28	0.68 (0.32-1.44)	33	0.62 (0.35-1.08)
	Day 28	28	1.48*(0.85-2.57)	33	1.05*(0.54-2.03)
	Month 6	27	1.59$^{\#}$(0.94-2.71)	30	0.99~$^{\#}$(0.51-1.93)
	**ARR**		**2.4 (1.4-4.0)**		**1.9 (1.4-2.4)**
	**AMR**		**0.93 (0.84-1.03)**		**0.88 (0.78-0.99)**
**Pn19F**	Day 0	28	0.99 (0.63-1.57)	33	0.92 (0.59-1.45)
	Day 28	28	2.49*(1.68-3.69)	33	1.56*(1.02-2.39)
	Month 6	27	2.36$^{\#}$(1.54-3.63)	30	1.28~$^{\#}$(0.79-2.08)
	**ARR**		**2.7 (1.8-4.1)**		**1.7 (1.3-2.2)**
	**AMR**		**0.92 (0.82-1.03)**		**0.81 (0.72-0.92)**
**Pn23F**	Day 0	28	0.60 (0.32-1.15)	33	0.34 (0.21-0.54)
	Day 28	28	1.86*(1.03-3.34)	33	0.67*(0.40-1.13)
	Month 6	27	1.43~$^{\#}$(0.78-2.60)	30	0.49~$^{\#}$(0.29-0.84)
	**ARR**		**3.4 (2.0-5.9)**		**2.0 (1.4-2.8)**
	**AMR**		**0.81 (0.70-0.94)**		**0.71 (0.60-0.83)**
**PPV23**	**ARR**		**3.6 (2.4-5.4)**		**2.4 (1.8-3.2)**
	**AMR**		**0.93 (0.87-1.00)**		**0.86 (0.79-0.93)**

Supplementary Figure 1

Older age is associated with poorer vaccine responses.
Both PPV23 ARR and TIV ARR show a significant negative correlation with age (**A,B** and **C,D** respectively) – panels **A** and **C** show statistics for the study population as a whole, panels **B** and **D** show the population split by disease group: CKD – blue and controls – white. Vaccine responders were significantly younger than non-reponders (**E**). NR – non-responder, R – responder (as defined in Methods); TIV – trivalent inactivated influenza vaccine; PPV23 – 23-valent pneumococcal polysaccharide vaccine; *Mann Whitney 2-tailed p<0.05 unless stated.

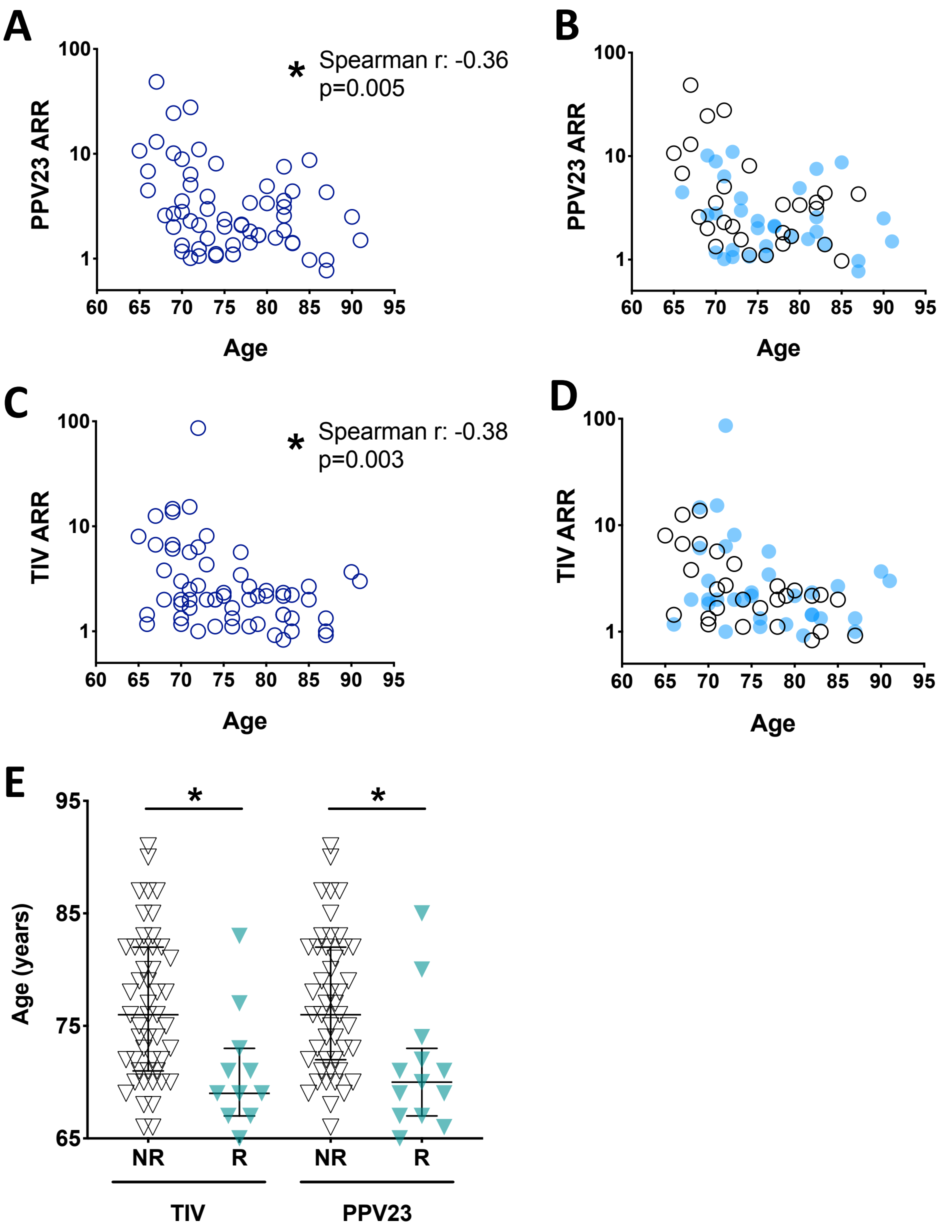

Supplementary Figure 2

Gating strategy for identification of T lymphocytes and subsequent subtyping (naïve/memory & "senescent" panels).

Singlet live T lymphocytes were identified by forward/side scatter characteristics, viability dye and surface expression of CD3 – scatter plots shown in **A**. Further sub-characterisation of T cell phenotype was then performed:

B: representative scatter plot for identification of CD4+ and CD8+ T cells.

C: naïve/memory subtypes identified by surface expression of CCR7 and CD45RA (representative density plots for CD4+ and CD8+ T cells shown).

D: representative density plot for CD28 and CD27 expression.

E: representative density plot for CD57 and KLRG1 expression.

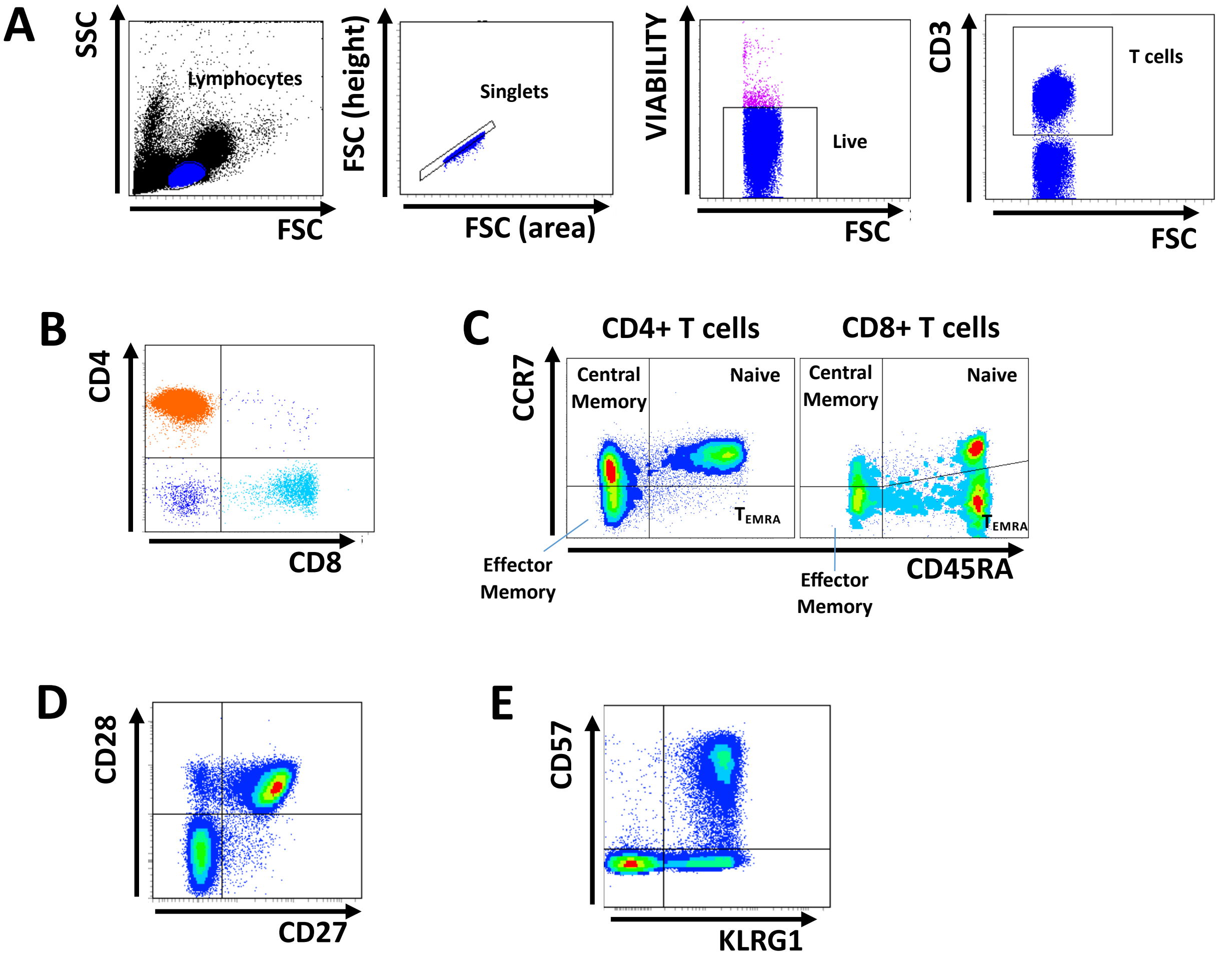

Supplementary Figure 3

Naïve/memory distribution of circulating T cells. Naïve/memory populations as defined using differential surface expression of CD45RA and CCR7 are shown for CD4$^+$ (**A-D**) and CD8$^+$ T cells (**E-H**), with comparison between controls (white) and patients with CKD (blue). CCR7$^+$CD45RA$^+$ - naïve, CCR7$^+$CD45RA$^-$ - central memory, CCR7$^-$CD45RA$^-$ - effector memory and CCR7$^-$CD45RA$^+$ - terminally differentiated effector memory cells (T_{EMRA}). Error bars denote median and IQR.

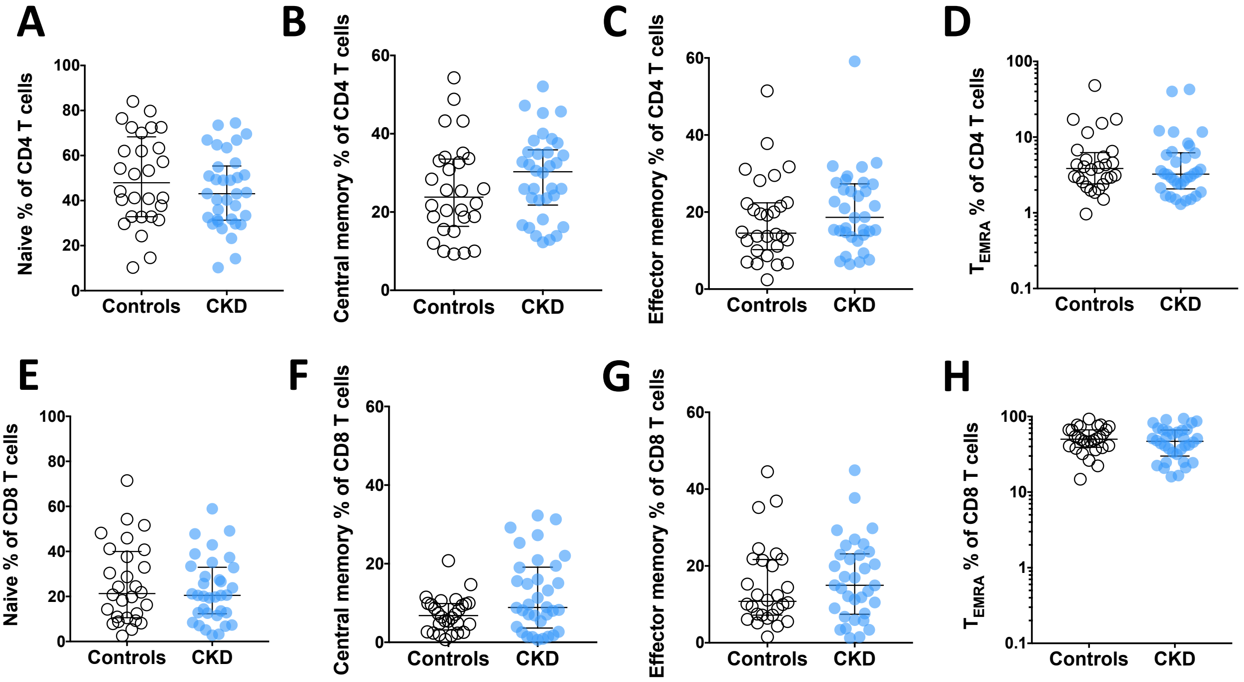

Supplementary Figure 4

“Senescent” CD8+ T cell phenotypes in patients with CKD and controls.

Comparisons of circulating “senescence”-associated $CD8^{+}$ populations are shown between controls and patients with CKD (**A-C**; CKD – blue, controls - white). Latent CMV infection was associated with expansions of these $CD8^{+}$ populations (**D-F**; CMV seropositive (+) – lilac, seronegative (-) – white). Error bars show median and IQR. *denotes Mann Whitney 2-tailed p<0.05.

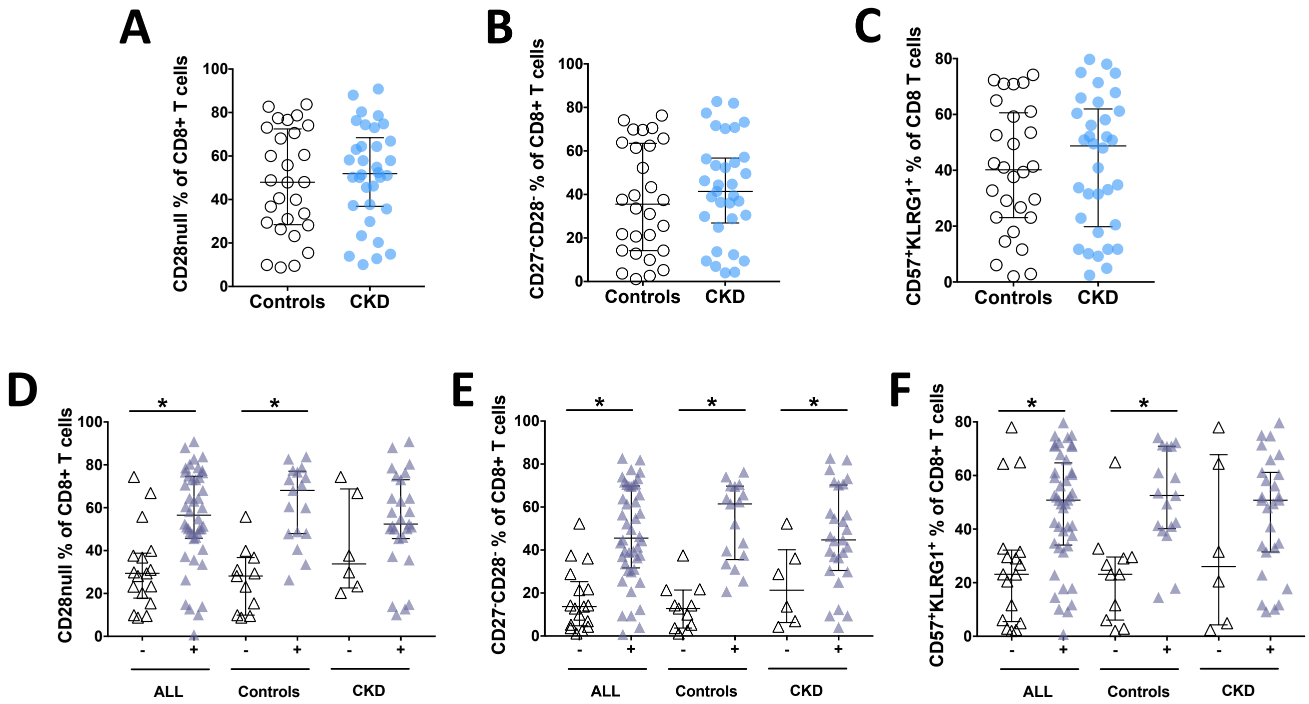

Supplementary Figure 5

Gating strategy for identification of CD4+ T lymphocyte subtypes.
Singlet live CD4+ T lymphocytes were identified by forward/side scatter characteristics, viability dye (as shown in Figure S2, A) and surface expression of CD3 and CD4 – scatter plot shown in **A**.
Further sub-characterisation of CD4+ T cell phenotype was then performed:
B: "Tfh-like" cells defined by CXCR5 expression (representative density plot shown).
C: Treg cells defined as $CD25^{high}FoxP3^{+}$ (highlighted in orange, representative scatter plot shown)
D: "Th1-", "Th2-", "Th17-" and "Th1Th17-like" cells defined by surface chemokine receptor expression as shown in representative density plots. CD4+ T cells were first evaluated for expression of CCR4 and CCR6. Each quadrant from this gate was subsequently evaluated for CXCR3 expression.
E, F: Th1 and Th2 subtypes defined by Tbet/IFN_{γ} and GATA3/IL-4 expression on stimulated PBMCs – representative scatter plots shown.

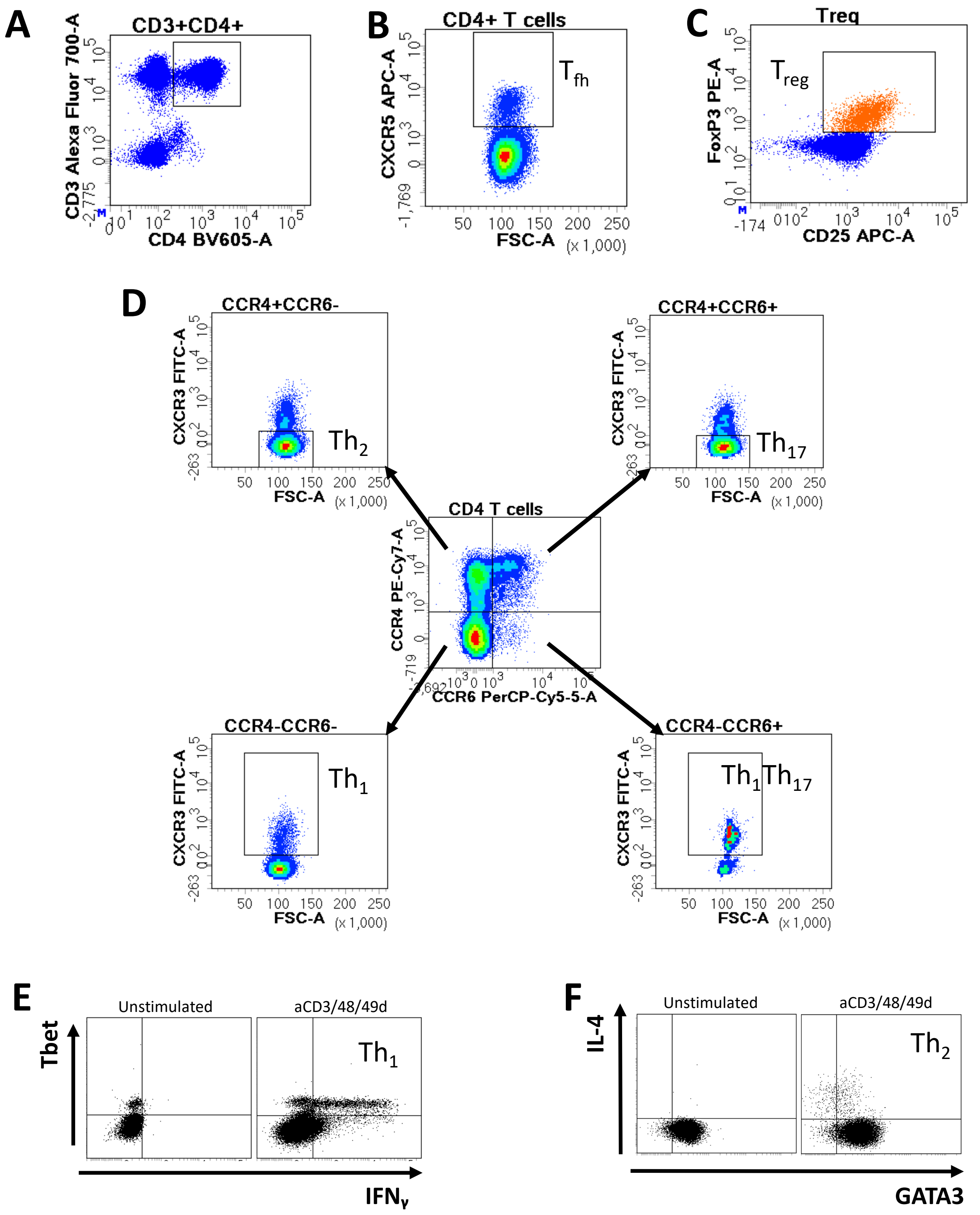

Supplementary Figure 6

Representative flow cytometry plots of $CD4^+$ T cell population differences between patients with CKD and controls. Representative results for comparison of Th_2 % of CD4 T cells between controls & CKD is shown in **A** (defined by surface chemokine receptor expression; “Th_2-like” population is gated as CCR4+ from CXCR3-CCR6- $CD4^+$ T cell population in this density plot for illustrative purposes) and **B** (defined as $CD4^+GATA3^+IL\text{-}4^+$ after overnight stimulation). Panel C shows representative density plots of T_{reg} % (defined as $CD4^+CD25^{high}$ $FoxP3^+$) in controls and patients with CKD.

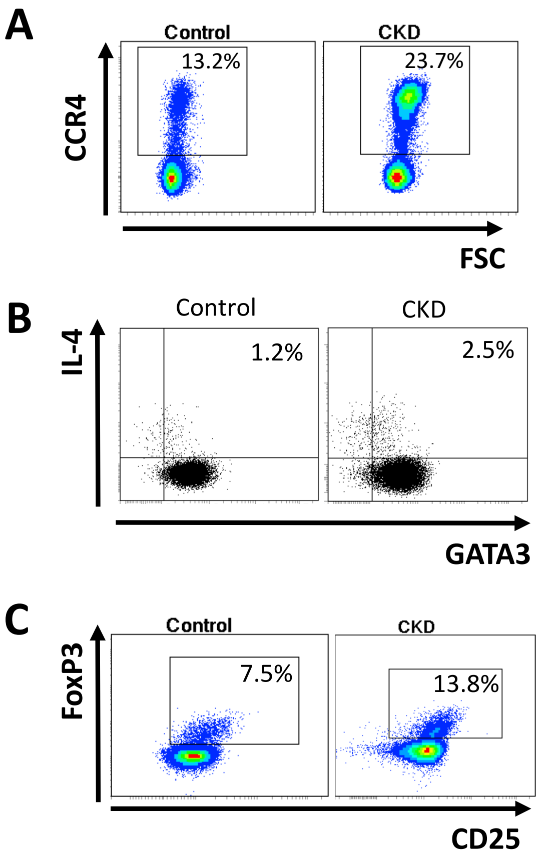

Supplementary Figure 7

B lymphocyte gating in patients with CKD and controls.
Singlet live B lymphocytes were identified by forward/side scatter characteristics, viability dye and surface expression of CD19 – scatter plots shown below.

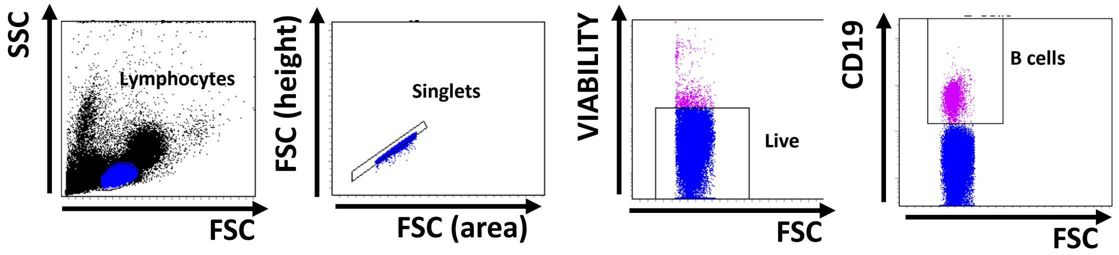

Supplement: ciab078_suppl_Supplementary_Data [file ciab078_suppl_supplementary_data.pdf]
